# The World Federation of Neurosurgical Societies Young Neurosurgeons Survey (Part II): Barriers to Professional Development and Service Delivery in Neurosurgery

**DOI:** 10.1016/j.wnsx.2020.100084

**Published:** 2020-05-11

**Authors:** Faith C. Robertson, Sujit Gnanakumar, Claire Karekezi, Kerry Vaughan, Roxanna M. Garcia, Bilal Abou El Ela Bourquin, Fahd Derkaoui Hassani, Alexander Alamri, Nesrine Mentri, Julius Höhne, Tsegazeab Laeke, Hosam Al-Jehani, Luis Rafael Moscote-Salazar, Ahmed Nasser Al-Ahmari, Nicolás Samprón, Martin N. Stienen, Federico Nicolosi, Davi J. Fontoura Solla, P. David Adelson, Franco Servadei, Amro Al-Habib, Ignatius Esene, Angelos G. Kolias, WFNS Young Neurosurgeons Committee

**Affiliations:** 1 Department of Neurosurgery, Massachusetts General Hospital, Boston, Massachusetts, USA; 2 School of Clinical Medicine, University of Cambridge, Cambridge, United Kingdom; 3 National Institute for Health Research Global Health Research Group on Neurotrauma, University of Cambridge, Cambridge, United Kingdom; 4 Department of Neurosurgery, Rwanda Military Hospital, Kigali, Rwanda; 5 Department of Neurosurgery, University of Pennsylvania, Philadelphia, Pennsylvania, USA; 6 Department of Neurosurgery, Northwestern University, Chicago, Illinois, USA; 7 Department of Neurosurgery, Cheikh Zaid International Hospital, Abulcasis International University of Health Sciences, Rabat, Morocco; 8 Department of Neurosurgery, The Royal London Hospital, London, United Kingdom; 9 Department of Neurosurgery, Bejaia University Hospital, Bejaia, Algeria; 10 Department of Neurosurgery, University Medical Center Regensburg, Regensburg, Germany; 11 Department of Surgery, Neurosurgery Unit, Addis Ababa University, College of Health Sciences, Addis Ababa, Ethiopia; 12 Department of Neurosurgery, King Fahad Hospital of the University, Imam Abdulrahman bin Faisal University, Al-Khobar, Saudi Arabia; 13 Neuroscience Center, King Fahad Specialist Hospital-Dammam, Dammam, Saudi Arabia; 14 Department of Neurosurgery, University of Cartagena, Cartagena de Indias, Colombia; 15 Division of Neurosurgery, Department of Neurosciences, King Faisal Specialist Hospital and Research Centre, Riyadh, Saudi Arabia; 16 Servicio de Neurocirugía, Hospital Universitario Donostia, San Sebastián, Spain; 17 Department of Neurosurgery, University Hospital Zurich & Clinical Neuroscience Center, University of Zurich, Switzerland; 18 Department of Neurosurgery, Humanitas University and Research Hospital, Rozzano, Milan, Italy; 19 Department of Neurosurgery, University of São Paulo, São Paulo, Brazil; 20 Barrow Neurological Institute at Phoenix Children’s Hospital, Phoenix, Arizona, USA; 21 Division of Neurosurgery, Department of Surgery, King Saud University, Riyadh, Saudi Arabia; 22 Neurosurgery Division, Department of Surgery, University of Bamenda, Bamenda, Cameroon; 23 Division of Neurosurgery, Department of Clinical Neurosciences, University of Cambridge and Addenbrooke’s Hospital, Cambridge, United Kingdom; 24 World Federation of Neurosurgical Societies Central Office, Nyon, Vaud, Switzerland

**Keywords:** Barriers, Capacity, Global health, Global neurosurgery, Neurosurgery, Service delivery, Training, 3D, Three-dimensional, CT, Computed tomography, HICs, High-income countries, LICs, Low-income countries, LMICs, Low-middle-income countries, MRI, Magnetic resonance imaging, QALYs, Quality-adjusted life years, UMICs, Upper-middle-income countries, WFNS, World Federation of Neurosurgical Societies

## Abstract

**Background:**

Strengthening health systems requires attention to workforce, training needs, and barriers to service delivery. The World Federation of Neurosurgical Societies Young Neurosurgeons Committee survey sought to identify challenges for residents, fellows, and consultants within 10 years of training.

**Methods:**

An online survey was distributed to various neurosurgical societies, personal contacts, and social media platforms (April–November 2018). Responses were grouped by World Bank income classification into high-income countries (HICs), upper middle-income countries (UMICs), low-middle-income countries (LMICs), and low-income countries (LICs). Descriptive statistical analysis was performed.

**Results:**

In total, 953 individuals completed the survey. For service delivery, the limited number of trained neurosurgeons was seen as a barrier for 12.5%, 29.8%, 69.2%, and 23.9% of respondents from HICs, UMICs, LMICs, and LICs, respectively (*P* < 0.0001). The most reported personal challenge was the lack of opportunities for research (HICs, 34.6%; UMICs, 57.5%; LMICs, 61.6%; and LICs, 61.5%; *P* = 0.03). Other differences by income class included limited access to advice from experienced/senior colleagues (*P* < 0.001), neurosurgical journals (*P* < 0.0001), and textbooks (*P* = 0.02). Assessing how the World Federation of Neurosurgical Societies could best help young neurosurgeons, the most frequent requests (*n* = 953; 1673 requests) were research (*n* = 384), education (*n* = 296), and subspecialty/fellowship training (*n* = 232). Skills courses and access to cadaver dissection laboratories were also heavily requested.

**Conclusions:**

Young neurosurgeons perceived that additional neurosurgeons are needed globally, especially in LICs and LMICs, and primarily requested additional resources for research and subspecialty training.

## Introduction

Health system strengthening for neurosurgery has continued to gain prominence in policy discussions and scientific literature as the global neurosurgical community strives to build capacity and improve timely access to safe and affordable neurosurgical care.^1^, ^2^, ^3^, ^4^ The advent of the Lancet Commission *Global Surgery 2030* report and the 2015 World Health Assembly Resolution 68.15 on emergency and essential surgery catalyzed investigations into the neurosurgical burden of disease and global workforce deficits.^1^^,^^2^^,^^4^, ^5^, ^6^, ^7^, ^8^, ^9^ For instance, the neurosurgical workforce is estimated to be around 50,000 neurosurgeons worldwide, but because of the burden of neurosurgical disease and unequal distribution of provider densities,^10^ many low-middle-income countries (LMICs) have a neurosurgical capacity of only 1%–10% of the minimum recommended neurosurgeon ratio per population, which is 0.01–0.1 neurosurgeons per 100,000 population.^11^, ^12^, ^13^ An excess of 23,000 more neurosurgeons are needed in LMICs to address the 5 million essential neurosurgical cases that go untreated each year.^6^ These untreated cases predominantly include traumatic brain injury but also incorporate stroke, hydrocephalus, tumors, epilepsy, and infection.^4^, ^5^, ^6^, ^7^, ^8^, ^9^ To address these issues, a systems-level approach is required.

The components of a health system, as outlined by the World Health Organization, include health service delivery, workforce, health information systems, access to essential medicines, financing, and leadership/governance.^14^ Within these 6 building blocks, there are many barriers that must be addressed to improve care provision. To expand the neurosurgical workforce, significant planning and investment are required to provide sufficient resources and methods of training for young neurosurgeons. However, variation in training needs across countries is not well understood. Elucidating the service delivery challenges for neurosurgical providers can inform future resource development and investments in supply management.

The World Federation of Neurosurgical Societies (WFNS) is committed to global improvement in neurosurgical care and recognizes that there is a paucity of studies that assess the needs of young neurosurgeons across economies. This cross-sectional survey performed by the WFNS Young Neurosurgeons Committee aimed to elucidate key needs of young neurosurgeons, their access to education and equipment, and the hurdles that they face in daily practice. The results presented report findings of 2 additional content areas not presented in Gnanakumar et al. (Part I), which includes perceptions on barriers and hurdles to deliver adequate neurosurgical care to local populations. These findings are intended to guide the structure of and investment in training programs to improve service delivery and facilitate timely access to safe and affordable neurosurgical care.

## Methods

### Survey Design, Dissemination, and Study Variables

The WFNS Young Neurosurgeons Committee aims to represent and promote the interests of young neurosurgeons, defined as residents, fellows, and consultants who are within 10 years of completing residency. The committee works to improve knowledge, surgical skills, research capability, and career opportunities for young neurosurgeons worldwide in alignment with the WFNS mission of benefiting patients and improving neurosurgical care.^15^

This cross-sectional study consisted of a Web-based survey performed between April 25 and November 30, 2018; details of the full methodology are as reported previously (Part I). This article focuses specifically on questions related to hurdles in daily practice and the personal needs of trainees. Respondents consisted of a nonprobabilistic sample of neurosurgeons invited though electronic mailing lists of continental and various neurosurgical societies, e-mail to personal contacts, and social media platforms (Twitter, Facebook, and WhatsApp).

### Statistical Analysis

Data were analyzed using commercially available software (SPSS version 25 [IBM Corp., Armonk, New York, USA] and Microsoft Excel 2016) to generate descriptive statistics. Responses were categorized according to the 2018 World Bank income classifications of high-income countries (HICs), upper-middle-income countries (UMICs), LMICs, and low-income countries (LICs).^16^ Descriptive statistical analysis included χ^2^ tests and analysis of variance for categorical and continuous variables, respectively. Multiple comparison adjustments were implemented if appropriate given survey question structure. Point estimates are presented with estimated 95% confidence intervals.

## Results

### Demographics

A total of 953 individuals completed the survey; completion was defined as 100% response to compulsory questions. Because of the wide dissemination of the questionnaire through social media platforms, calculation of a response rate was not possible. Categorized according to World Bank income classifications, there were 431 respondents from HICs, 228 from UMICs, 255 from LMICs, and 39 from LICs. A more detailed examination of the respondents’ demographics, scope of clinical practice, and nuances in access to training and equipment resources (e.g., computed tomography [CT] or magnetic resonance imaging) is reported in a separate report by Gnanakumar et al. (Part I).

### Barriers in Delivering an Adequate Neurosurgical Service

About one quarter of global respondents (25.8%) identified that local neurosurgical needs were adequately met (Table 1). There was a graduated reduction from 38.8% in HICs to 10.3% in LICs (*P* < 0.0001). More than half of respondents in LMICs and LICs reported inadequate or no insurance coverage for many people. The limited number of trained neurosurgeons was seen as a barrier for 12.5%, 29.8%, 69.2%, and 23.9% of respondents from HICs, UMICs, LMICs, and LICs, respectively (*P* < 0.0001). Similar patterns were seen for limitations arising from a dearth of space and resources. More than 30% of individuals from UMICs and LMICs, and >50% from LICs, expressed that the paucity of neurosurgical beds was a barrier to care delivery (*P* < 0.001), and >40% of respondents from UMICs and LMICs, and >50% from LICs reported challenges regarding intensive care unit beds (*P* < 0.01).

**Table 1:** Perceived Systemic Barriers to Meeting the Needs of the Local Population

	High-Income Economies (n = 431), n (%) (95% CI)	Upper-Middle-Income Economies (n = 228), n (%) (95% CI)	Lower-Middle-Income Economies (n = 255), n (%) (95% CI)	Low-Income Economies (n = 39), n (%) (95% CI)	Total (n = 953), n (%) (95% CI)	*P* Value
N/A–the neurosurgical care needs of my local population are perfectly covered	167 (38.8) (34.3–43.4)	47 (20.6) (15.9–26.3)	28 (11) (7.7–15.4)	4 (10.3) (4.1–23.6)	246 (25.8) (23.1–28.7)	<0.0001
Inadequate or no insurance coverage for significant number of people	36 (8.4) (6.1–11.3)	70 (30.7) (25.1–37)	150 (58.8) (52.7–64.7)	21 (53.9) (38.6–68.4)	277 (29.1) (26.3–32)	<0.0001
The limited number of trained neurosurgeons	54 (12.5) (9.7–16)	68 (29.8) (24.3–36.1)	79 (31) (25.6–36.9)	27 (69.2) (53.6–81.4)	228 (23.9) (21.3–26.7)	<0.0001
The limited number of neurosurgical beds	92 (21.4) (17.7–25.5)	75 (32.9) (27.1–39.2)	78 (30.6) (25.3–36.5)	21 (53.9) (38.6–68.4)	266 (27.9) (25.2–30.8)	<0.001
The limited number of intensive care unit beds	104 (24.1) (20.3–28.4)	99 (43.4) (37.2–49.9)	124 (48.6) (42.6–54.7)	21 (53.9) (38.6–68.4)	348 (36.5) (33.5–39.6)	0.01
Lack of access to equipment necessary for microsurgery (e.g. microscope, drill, bipolar)	22 (5.1) (3.4–7.6)	70 (30.7) (25.1–37)	117 (45.9) (39.9–52)	21 (53.9) (38.6–68.4)	230 (24.1) (21.5–27)	<0.0001
Lack of regular/consistent access to computed tomography	5 (1.2) (0.5–2.7)	8 (3.5) (1.8–6.8)	29 (11.4) (8–15.9)	10 (25.6) (14.6–41.1)	52 (5.5) (4.2–7.1)	<0.0001
Lack of regular access to magnetic resonance imaging	30 (7) (4.9–9.8)	50 (21.9) (17.1–27.7)	55 (21.6) (17–27)	18 (46.2) (31.6–61.4)	153 (16.1) (13.9–18.5)	<0.0001
Lack of organized primary care	52 (12.1) (9.3–15.5)	58 (25.4) (20.2–31.5)	87 (34.1) (28.6–40.1)	10 (25.6) (14.6–41.1)	207 (21.7) (19.2–24.5)	0.02
Lack of organized prehospital/emergency hospital care	42 (9.7) (7.3–12.9)	59 (25.9) (20.6–31.9)	108 (42.4) (36.4–48.5)	21 (53.9) (38.6–68.4)	230 (24.1) (21.5–27)	<0.0001
Lack of organized rehabilitation care	79 (18.3) (15–22.3)	76 (33.3) (27.5–39.7)	105 (41.2) (35.3–47.3)	21 (53.9) (38.6–68.4)	281 (29.5) (26.7–32.5)	<0.001
Other	63 (14.6) (11.6–18.3)	16 (7) (4.4–11.1)	20 (7.8) (5.1–11.8)	4 (10.3) (4.1–23.6)	103 (10.8) (9–12.9)	0.2839

Summary of young neurosurgery respondents' (n = 953) perceived systemic barriers to meeting the needs of the local population by World Bank income classification.

CI, confidence interval.

Perceived access to essential imaging modalities was another barrier associated with significant differences across country income classes. Among LICs, 25.6% of respondents identified challenges in CT accessibility (*P* < 0.0001) and 46.2% for magnetic resonance imaging (*P* < 0.0001). Regarding equipment, lack of access to tools such as microscopes, high-speed drills, or bipolar cautery was identified as a barrier by 5.1%, 30.7%, 45.9%, and 53.9% of respondents from HICs, UMICs, LMICs, and LICs, respectively (*P* < 0.0001).

Relating to the spectrum of care, limitations in organized primary care were respectively highlighted as barriers by 12.1%, 25.4%, 34.1%, and 25.6% of HICs, UMICs, LMICs, and LICs respondents (*P* = 0.02). A lack of organized prehospital and emergency hospital care was identified by 9.7%, 25.9%, 42.4%, and 53.9% of those from HICs, UMICs, LMICs, and LICs (*P* < 0.0001); an analogous trend was evident for organized rehabilitation care (*P* < 0.001). Overall, increased hurdles endured by those practicing in LICs was further shown by the fact that respondents identified on average 1.34 hurdles impeding their practice in HICs, compared with an average of 5.0 for LICs (analysis of variance, *P* < 0.05, with Bonferroni correction showing significant difference between HICs and both LMICs [*P* < 0.003] and LICs [*P* < 0.001] but not UMICs [*P* = 0.136]).

### Barriers in Personal Practice

A similar pattern emerged related to personal barriers encountered during daily care provision (Table 2). The most common reported challenge identified was limited opportunities to conduct research (total, 48.4%; 34.6% for HICs; 57.5% for UMICs; 61.6% for LMICs; and 61.5% for LICs; *P* = 0.03). Other significant differences observed in barriers associated with income class included lack of regular access to the advice of experienced/senior colleagues (12.3%, 22.4%, 21.2%, and 41.0% of individuals from HICs, UMICs, LMICs, and LICs, respectively; *P* < 0.001), lack of access to neurosurgical journals (11.8%, 26.3%, 25.1%, and 64.1% of individuals from HICs, UMICs, LMICs, and LICs, respectively; *P* < 0.0001), and lack of access to neurosurgical textbooks (7.4%, 16.2%, 17.3%, and 25.6% from HICs, UMICs, LMICs, and LICs, respectively; *P* = 0.02). Barriers that were similar across income groups included access to a mentor (>24% for all; highest in LICs, 38.5%), lack of hands-on opportunities for surgical training (average, 44.6%; highest in LICs, 56.4%) and organized teaching/training sessions (average, 44.6%; highest in LICs, 51.3%). Regarding working conditions and culture, 41.6% of individuals listed long work hours as a challenge, and 40.9% noted poor work–life balance, and 13.2% reported bullying and harassment issues; these obstacles were present across all income groups. Similar to the hurdles affecting local provision of care, HICs respondents reported an average of 2.6 issues, whereas neurosurgeons in LICs reported 4.5 (*P* = 0.86).

**Table 2:** Perceived Personal Challenges Encountered in Daily Practice

	High-Income Economies (n = 431), n (%) (95% CI)	Upper-Middle-Income Economies (n = 228), n (%) (95% CI)	Lower-Middle-Income Economies (n = 255), n (%) (95% CI)	Low-Income Economies (n = 39), n (%) (95% CI)	Total (n = 953), n (%) (95% CI)	*P* Values
N/A–there are no hurdles	56 (13) (10.1–16.5)	14 (6.1) (3.7–10)	8 (3.1) (1.6–6.1)	1 (2.6) (0.5–13.2)	79 (8.3) (6.7–10.2)	0.01
Lack of access to organized teaching/training sessions	157 (36.4) (32–41.1)	113 (49.6) (43.1–56)	126 (49.4) (43.3–55.5)	20 (51.3) (36.2–66.1)	416 (43.7) (40.5–46.8)	0.35
Limited number of opportunities for hands-on operating	187 (43.4) (38.8–48.1)	100 (43.9) (37.6–50.4)	116 (45.5) (39.5–51.6)	22 (56.4) (41–70.7)	425 (44.6) (41.5–47.8)	0.52
Long hours of work	162 (37.6) (33.2–42.3)	107 (46.9) (40.6–53.4)	111 (43.5) (37.6–49.7)	16 (41) (27.1–56.6)	396 (41.6) (38.5–44.7)	0.79
Poor work/life balance	153 (35.5) (31.1–40.1)	97 (42.5) (36.3–49)	122 (47.8) (41.8–54)	18 (46.2) (31.6–61.4)	390 (40.9) (37.8–44.1)	0.59
Bullying and harassment issues	53 (12.3) (9.5–15.7)	33 (14.5) (10.5–19.6)	36 (14.1) (10.4–18.9)	4 (10.3) (4.1–23.6)	126 (13.2) (11.2–15.5)	0.76
Lack of regular access to the advice of experienced/senior colleagues	53 (12.3) (9.5–15.7)	51 (22.4) (17.4–28.2)	54 (21.2) (16.6–26.6)	16 (41) (27.1–56.6)	174 (18.3) (15.9–20.8)	<0.001
Lack of a mentor	110 (25.5) (21.6–29.8)	55 (24.1) (19–30.1)	65 (25.5) (20.5–31.2)	15 (38.5) (24.9–54.1)	245 (25.7) (23–28.6)	0.17
Lack of access to neurosurgical journals	51 (11.8) (9.1–15.2)	60 (26.3) (21–32.4)	84 (25.1) (20.2–30.8)	25 (64.1) (48.4–77.3)	220 (28.6) (20.5–25.9)	<0.0001
Lack of access to neurosurgical textbooks	32 (7.4) (5.3–10.3)	37 (16.2) (12–21.6)	44 (17.3) (13.1–22.4)	10 (25.6) (14.6–41.1)	123 (12.9) (10.9–15.2)	0.02
Limited opportunities to do research	149 (34.6) (30.2–39.2)	131 (57.5) (51–63.7)	157 (61.6) (55.5–67.3)	24 (61.5) (45.9–75.1)	461 (48.4) (45.2–51.5)	0.03
Other	25 (5.8) (4–8.4)	10 (4.4) (2.4–7.9)	16 (6.3) (3.9–9.9)	4 (10.3) (4.1–23.6)	55 (5.8) (4.5–7.4)	0.40

Summary of young neurosurgery respondents (*n* = 953) perceived personal challenges encountered in daily practice by World Bank income classification.

CI, confidence interval.

### Requested Areas of Improvement

**Figure 1:**
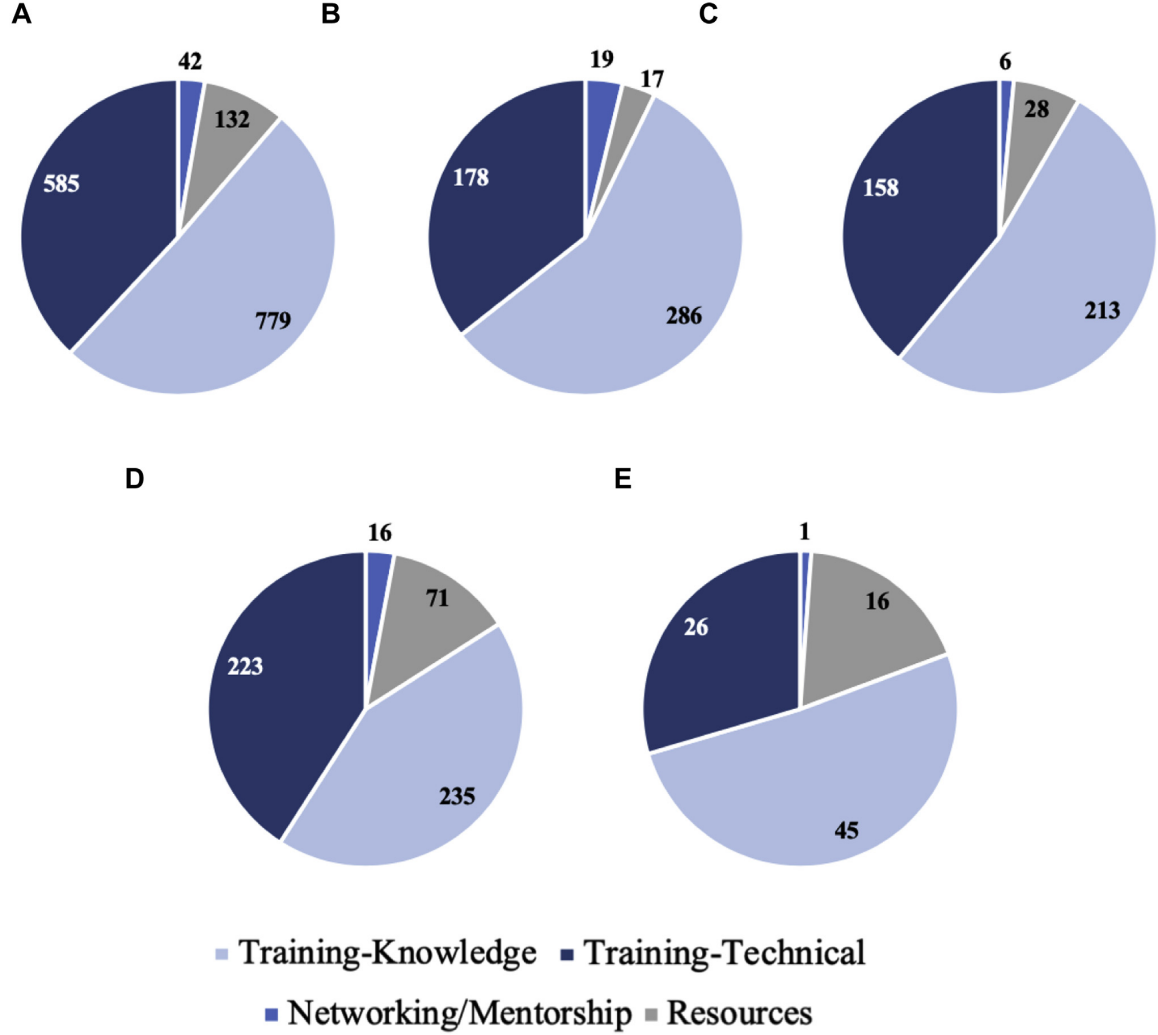
Categorization of respondent requests into categories of knowledge-based training, technical training, networking/mentorship, and resources. (**A**) Overall respondents and (**B–E**) by World Bank income classification: (**B**) high-income countries, (**C**) upper middle-income countries, (**D**) low-middle-income countries, and (**E**) low-income countries.

**Figure 2:**
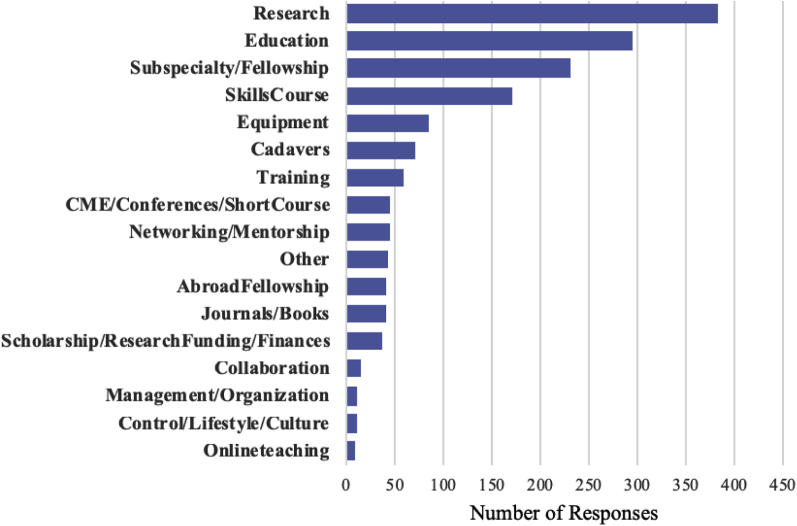
Detailed categorization of respondents' requests for improvement in their current neurosurgical system. Of the 1673 individual requests for system improvement, the most frequent request was for research (384 individuals), followed by additional education (296 individuals), and additional subspecialty or fellowship training requests (232 respondents). The subspecialties of interest are shown below. Twenty-five percent of fellowship requests came from high-income countries, 26.3% from upper-middle-income countries, 44.0% from low-middle-income countries, and 4.74% from low-income countries. CME, continuing medical education.

When asked to list 3 areas in which the WFNS could facilitate the respondent’s personal goals and the goals of their neurosurgical service, there were 1673 responses from 953 individuals. Results span categories of system improvement, education, and technical training. Figure 1 shows broad categories of knowledge-based training, technical training, networking/mentorship, and resources, by income class, and Figure 2 shows the overall detailed responses.

**Figure 3:**
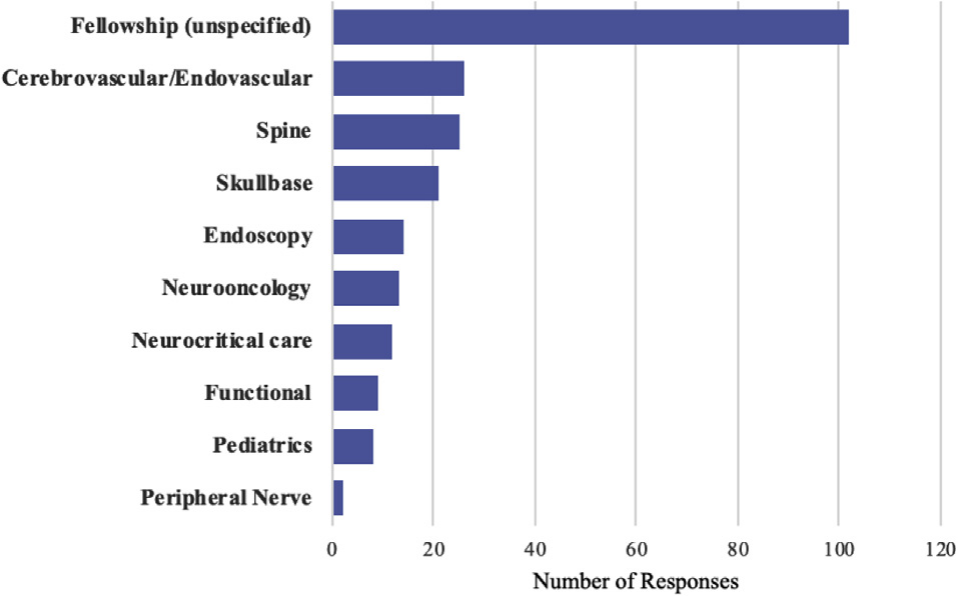
Requested fellowships from young neurosurgery respondents. A total of 232 individuals expressed interest in additional fellowship training. Of the specified fields (102 unspecified), most respondents requested training in cerebrovascular (*n* = 26), spine (*n* = 25) and skull base (*n* = 21).

Of the 1673 individual requests for improvement, the most frequent request was for research (384 individuals, 40.3%), followed by additional education opportunities (296 individuals, 31.1%), and additional subspecialty or fellowship training requests (232 respondents, 24.3%). Specific subspecialties of interest are shown in Figure 3. Of those who mentioned a specific subspecialty (130/232), most requested training in cerebrovascular (*n* = 26), spine (*n* = 25), and skull base (*n* = 21). Regarding nontechnical training, many individuals requested additional venues to continue medical education through courses and conferences or online courses. For technical training, there were 171 and 71 requests for skills courses/workshops and cadaver dissection opportunities, respectively.

## Discussion

This survey is the most current and, to our knowledge, the most comprehensive cross-sectional examination of the global barriers that young neurosurgeons encounter during neurosurgical training and service delivery. It is critical that the global neurosurgery community is aware of these challenges so there can be a systematic response to empower the neurosurgical workforce and mitigate the global burden of neurosurgical disease.^4^ Overall, the factors that individuals identified as barriers to optimal training and care provision closely mirrored the requests to the WFNS Young Neurosurgeons Committee for improvement in the following sections. They can be categorized into desired improvements in resources for service delivery, neurosurgical education (nontechnical skills), and continued development of technical skills. Current efforts and opportunities for future investment are described.

### Service Delivery

The challenges in service delivery span the spectrum of health care delivery, and respondents identified these barriers arising from primary care, emergency services, hospital bed availability, and rehabilitation. Even respondents from HICs desired access to more beds, although this finding does not account for the significant differences in baseline bed numbers. These hurdles necessitate tremendous investment in infrastructure at every level. For this reason, there was a recent development of the *Comprehensive Policy Recommendations for Head and Spine Injury Care in LMICs*.^17^ This document focuses on emergency care, but investing in trauma infrastructure enables improvements in the flow of elective cases as well. The recommendations span neurotrauma surveillance, prevention, prehospital care, hospital care, and rehabilitation stages and it discusses all in the context of infrastructure, workforce, service delivery, financing, information management, and governance.

The scarcity of equipment for procedures was another major obstacle. The WFNS Foundation is working with medical equipment sponsors to provide high-quality neurosurgical equipment at an affordable cost to neurosurgeons in economically challenged countries who are devoted to neurosurgery and their patients. As of December 2018, the WFNS Foundation has dispatched 58 neurosurgical kits to Asia and Australasia, 16 neurosurgical kits to the Middle East, 24 neurosurgical kits to Europe, 18 neurosurgical kits to Latin America, and 125 neurosurgical kits to Africa.^18^ Although equipment donations will advance care in the short term, local health systems are called to invest in sustainable resource support. In addition, innovation in low-cost devices and procedures can improve long-term cost-effectiveness. For example, the University of Cape Town, South Africa, developed the Cape Town Stereotactic Pointer as a low-cost simple device to obviate the use of frames and devices associated with traditional stereotactic techniques.^19^ Handheld near-infrared spectroscopy devices are being increasingly used to triage and diagnose patients with intracranial hematomas, which can be a vital tool when or where CT scanners are unavailable.^20^ We believe that neurosurgeons will need to continue partnering with engineers, industry, and other disciplines to further the development of low-cost innovation for neurosurgical care delivery.

### Neurosurgical Education (Nontechnical Skills)

Our survey shows strong interest among trainees in research opportunities. Strengthening networks between local and national or international centers is needed to create opportunities for local trainee involvement. On the WFNS Web site there are multiple postings for clinical and research observers and fellows; trainees are encouraged to apply and universities are encouraged to continue funding these efforts.^21^ In addition, large collaborative studies that invite global participation are increasing in prevalence. A recent example is the National Institute for Health Research Unit on Global Surgery’s establishment of transnational research hubs to coordinate surgical research, including conducting international randomized clinical trials.^22^ Specific to neurosurgery, the National Institute for Health Research Global Health Research Group on Neurotrauma, hosted at the University of Cambridge, United Kingdom is conducting a prospective multicenter international cohort study of outcomes after emergency surgery for traumatic brain injury, in which local trainees can contribute to data collection on outcomes and follow-up both before and after intervention (Global Neurotrauma Outcomes Study).^23^ Moreover, these initiatives can provide funding for trainees who wish to contribute more by undertaking Ph.D. research. The same group has a specific theme that aims to nurture the traumatic brain injury research capacity in LMICs.^24^ The group is facilitating this initiative with the funding of 1) research fellow posts in each participating institution, 2) exchanges between institutions, and 3) courses focused on clinical care and research methodology. InterSurgeon is another free service that brings together neurosurgeons who wish to collaborate in clinical practice, participate in the provision of training and education, or share equipment and other resources.^25^

Barriers to access to journals, particularly in LICs, were raised as an impediment to personal development. Major impactful neurosurgical articles are published in journals such as *Journal of Neurosurgery*, *Neurosurgery*, *Acta Neurochirurgica (Wien)*, and *World Neurosurgery*, but paywalls and requisites for individual subscriptions can cost hundreds of dollars per annum. For young neurosurgeons in LICs and LMICs, this can be the equivalent of more than a month’s salary. Therefore, we invite open-access publication initiatives such as those in which authors pay toward the cost of making articles accessible for free. Many research funders, including United Kingdom Research Councils and the Wellcome Trust, already require funded work to be made open access after an embargo period.^26^^,^^27^ Although many LMICs and LICs researchers may not be able to afford the article-processing charge, additional grants for these researchers to open-access publishing should be considered.^28^ Other initiatives include offering access to journals to researchers in developing countries at reduced or no cost.^29^ Overall, the neurosurgical community should make a concerted effort to increase the accessibility of research articles to young neurosurgeons in LMIC and LICs. In addition, the WFNS continues to support and broadcast opportunities for learning such as live surgery seminars and educational courses, which can be found at https://wfns.org/events. The WFNS Young Neurosurgeons Committee has also initiated a series of monthly webinars, which become immediately and permanently available to all on YouTube. Although we acknowledge that access to reliable Internet remains a challenge for many young neurosurgeons, there is constant advancement in the ease and affordability of accessing online material through smartphone and computer data, and this remains one of the most rapid and practical means of information dissemination.

### Technical Skills and Fellowships

The survey elucidated the unmet need for additional technical training opportunities, with particular interest in technical skills workshops, cadaver laboratories, and clinical fellowship. Because cadavers can be costly and difficult to obtain, low-cost simulation models may be a great solution.^30^^,^^31^ For instance, a recent study on subspecialty pediatric neurosurgery training reported a low-cost skill-based training model for neurosurgeons in low-resourced health systems. Trainees were oriented to an endoscopic simulation station outfitted with cranial models of infants with hydrocephalus, and each cranial model, designed from thin-cut radiographs, was three-dimensionally (3D) printed at a cost of approximately U.S. $4.^31^ As 3D printing quality improves and cost declines, neurosurgical model development for training is encouraged. In addition, the WFNS is continuing to work to offer regional skills training workshops. The European Association of Neurosurgical Societies and AO Spine offer high-quality training courses, albeit priced at >$1000 each; solutions could be to decrease fees for participants from LICs or offer additional regional courses with support from industry and WFNS. The WFNS Young Neurosurgeons Committee has also partnered with UpSurgeOn, a multidisciplinary team of neurosurgeons, developers, digital artists, and artisans that envisioned a revolution of head, neck, otolaryngology, and spine surgery training using hi-tech/low-cost technology. This initiative intends to bridge the gap between theoretic learning and practical training through physical models fused with augmented reality 3D models for psychomotor skill training using hybrid solutions. The UpSurgeOn technologies, like AppSurgeOn Apps and UpSim Neurosurgical Box, have been designed to be affordable also for training in countries with limited facilities. Since March 2018, AppSurgeOn Apps has hosted a real-time stream dedicated to WFNS Young Neurosurgeons activities. The stream is able to reach around half a million users worldwide.

The most requested subspecialty fellowships were cerebrovascular and spine. The global burden of stroke and the paucity of angiography in lower-income settings may be driving the cerebrovascular interest, but our survey did not distinguish between open versus endovascular training. However, it is important to consider both the epidemiology of disease and the cost-effectiveness of cerebrovascular interventions. In a study estimating the economic consequences of neurosurgical disease in LMICs,^32^ most of the losses were attributed to stroke and traumatic brain injury. However, in a cost-effectiveness analysis of mechanical thrombectomy in China, the addition of mechanical thrombectomy to intravenous tissue plasminogen activator treatment compared with standard treatment alone yielded a lifetime gain of 0.794 quality-adjusted life years (QALYs) or U.S. $9690 per QALY gained.^33^ The probabilistic sensitivity analysis was run with a willingness-to-pay threshold of U.S. $19,300 per QALY. Few respondents identified additional interests in pediatrics training, despite the large global burden of congenital conditions and hydrocephalus,^7^ and the cost per disability adjusted life years averted ranges from U.S. $59 to $126.^34^ Furthermore, only approximately 330 pediatric neurosurgeons are taxed with caring for a population of 1.2 billion children.^5^^,^^35^ There should be positive incentives for trainees to specialize in pediatric neurosurgery. Investing in subspecialty training should incorporate both the population need, based on disease burden, as well as cost-effectiveness strategies and should be integrated into infrastructure development.^36^

The WFNS Office of Training Centers & Fellowship orchestrates fellowships at 23 postgraduate, 2 short-term, and 4 full-program training centers. These are based worldwide and include Brazil, China, Malaysia, Morocco, Germany, Greece, France, India, Indonesia, Italy, Japan, Jordan, Pakistan, Singapore, South Africa, Spain, United Kingdom, and United States.^37^ For these fellowships, the trainee is provided with a stipend for food and accommodation. The WFNS–Rabat Training Center with a faculty of 29 professors and teachers has trained 58 young neurosurgeons from 18 sub-Saharan African countries over an 18-year period (2002–2019).^38^ Thirty of these neurosurgeons have finished their training and returned home to practice and teach neurosurgery in public hospitals. As part of its commitment to continuing medical education, the Center also organizes 3 courses and workshops every year. Initiatives such as CURE Hydrocephalus and Spina Bifida offer subspecialty fellowships to neurosurgeons from LICs, allowing these young trainees to pursue their subspecialty interests. The Ethiopian partnership with the Norwegian University of Bergen and Foundation for International Education in Neurological Surgery facilitated an increase in neurosurgical capacity from 2 neurosurgeons in 2006 to 30 in 2019. Recently, a new East African training program was created in collaboration with the College of Surgeons of East Central and Southern African, with training sites in Tanzania, Uganda, Kenya, and Ethiopia. Programs in Senegal, Zimbabwe, and South Africa are also actively expanding their neurosurgical workforce. The benefits of these programs are that they are sensitive to the local context of culture, disease, and resource availability and increase the likelihood of trainees to stay in their home countries and build neurosurgical capacity.

### Future Directions

The WFNS will take these data into account as they advocate for investment in resources and education for young neurosurgeons. In addition, neurosurgeons from HICs can partner with LICs as they begin to formulate their national surgical plans and strive to address the burden of neurosurgical disease in their respective countries. Sustainable partnerships between neurosurgery departments in HICs and LICs should continue to be developed to create opportunities for training, mentoring, and research, particularly in sub-Saharan Africa, Southeast Asia, and Latin America. Professional national and regional neurosurgical societies have an opportunity to support their local communities of neurosurgeons to deliver high-quality neurosurgical care via continuing surgical education, surgeon fellowship, peer evaluation, scientific exchange, organizing manpower and funding for international initiatives, developing practice guidelines, and lobbying for federal support. These societies can provide a springboard from which to launch targeted interventions, including research, at a local level. We encourage young neurosurgeons to stay connected to the WFNS to seek out resources and opportunities as they arise, and we call on the global neurosurgical community to come together in these efforts.

### Limitations

The major limitations of this study include issues related to convenience sampling methodologies that precluded response rate calculation, and the opinions of those without reliable Internet, electronic devices, and e-mail are less likely to be captured. Administering the survey in English limited respondents to those with sufficient English comprehension. Young neurosurgeons from many geographic areas, especially East Asia and Pacific, were not adequately represented; this situation may have resulted from survey distribution, language barriers, or other unknown factors. More goal-directed studies are needed in the future to capture these populations. Approximately 60% of respondents were from cities of >0.5 million people, and >80% were from cities with populations >200,000, thus representing young neurosurgeons and trainees in more urban areas. However, this situation is also indicative of the nature of neurosurgical practice, in which multiple surgeons are often clustered in urban centers. The role played by academic and research contacts in dissemination of the survey may have introduced selection bias, particularly pertaining to the question regarding payment for clinical work versus research; >20% of respondents reportedly receiving payment for research, and it was the top request for improvement in their current neurosurgical system. Although there are still country-specific and hospital-specific needs that need to be addressed on a more country-specific and region-specific level to understand unique factors, this survey provides a broad overview of barriers to training and service delivery for young neurosurgeons and can serve as a guide for resource strategies, partnership development, and system improvement.

## Conclusions

This global survey aimed to elucidate challenges faced by young neurosurgeons across economies. It showed key health system barriers that can be improved with the development of national surgical plans, partnerships, and resource investments. It also underscored which areas of nontechnical and technical skill development are a priority for young neurosurgeons, such as opportunities for research, access to peer review publications, skills-based workshops with cadavers or models, and desired fields of subspecialty training. Although the WFNS will continue to work to improve these areas, we call on the global neurosurgical community to partner with us in these efforts.

## Declaration of Competing Interest

A.G.K. is supported by the 10.13039/501100000272National Institute for Health Research (NIHR) Global Health Research Group on Neurotrauma. The Group was commissioned by the 10.13039/100006662NIHR using UK aid from the UK Government (project 16/137/105). The views expressed in this article are those of the authors and are not necessarily those of the United Kingdom National Health Service, NIHR, or the United Kingdom Department of Health. F.N. is Founder and Chief Executive Officer of UpSurgeOn, viale Monza 347, 20126 Milan. R.M.G. serves as a National Institute of Health (NIH) Fogarty Global Health Fellow and Scholar. Research reported in this publication was supported by the 10.13039/100000061Fogarty International Center and 10.13039/100000025National Institute of Mental Health, of the 10.13039/100000002National Institutes of Health under Award Number D43TW010543. The content is solely the responsibility of the authors and does not necessarily represent the official views of the National Institutes of Health.

## CRediT authorship contribution statement

**Faith C. Robertson:** Methodology, Investigation, Data curation, Writing - original draft. **Sujit Gnanakumar:** Methodology, Investigation, Data curation, Writing - original draft. **Claire Karekezi:** Conceptualization, Writing - review & editing. **Kerry Vaughan:** Conceptualization, Writing - review & editing. **Roxanna M. Garcia:** Conceptualization, Writing - review & editing. **Bilal Abou El Ela Bourquin:** Methodology, Investigation, Data curation, Writing - original draft. **Fahd Derkaoui Hassani:** Conceptualization, Writing - review & editing. **Alexander Alamri:** Conceptualization, Writing - review & editing. **Nesrine Mentri:** Conceptualization, Writing - review & editing. **Julius Höhne:** Conceptualization, Writing - review & editing. **Tsegazeab Laeke:** Conceptualization, Writing - review & editing. **Hosam Al-Jehani:** Conceptualization. **Luis Rafael Moscote-Salazar:** Conceptualization, Writing - review & editing. **Ahmed Nasser Al-Ahmari:** Conceptualization, Writing - review & editing. **Nicolás Samprón:** Conceptualization, Writing - review & editing. **Martin N. Stienen:** Writing - review & editing. **Federico Nicolosi:** Conceptualization. **Davi J. Fontoura Solla:** Conceptualization, Data curation, Writing - review & editing. **P. David Adelson:** Conceptualization, Writing - review & editing. **Franco Servadei:** Conceptualization, Writing - review & editing. **Amro Al-Habib:** Conceptualization, Writing - review & editing. **Ignatius Esene:** Conceptualization, Data curation, Writing - review & editing. **Angelos G. Kolias:** Conceptualization, Methodology, Investigation, Data curation, Writing - review & editing.
